# COVID-19 and mental well-being of nurses in a tertiary facility in Kenya

**DOI:** 10.1371/journal.pone.0254074

**Published:** 2021-07-01

**Authors:** Sayed K. Ali, Jasmit Shah, Zohray Talib

**Affiliations:** 1 Department of Medicine, Aga Khan University Hospital, Nairobi, Kenya; 2 Department of Medical Education, California University of Science and Medicine, Colton, California, United States of America; Endeavour College of Natural Health, AUSTRALIA

## Abstract

**Background:**

The 2019 coronavirus disease (COVID-19) epidemic is a global health emergency which has been shown to pose a great challenge to mental health, well-being and resilience of healthcare workers, especially nurses. Little is known on the impact of COVID-19 among nurses in sub-Saharan Africa.

**Methods:**

A cross sectional study was carried out between August and November 2020 among nurses recruited from the Aga Khan University Hospital, Nairobi. The survey questionnaire consisted of six components- demographic and work title characteristics, information regarding care of COVID-19 patients, symptoms of depression, anxiety, insomnia, distress and burnout, measured using standardized questionnaires. Multivariable logistic regression analysis was performed to identify factors associated with mental health disorders.

**Results:**

Of 255 nurses, 171 (67.1%) consented to complete the survey. The median age of the participants was 33.47 years, 70.2% were females and 60.8% were married. More than half, 64.9% were frontline workers directly engaged in COVID-19 care. Only 1.8% reported a prior history or diagnosis of any mental health disorder. Depression, anxiety, insomnia, distress, and burnout were reported in 45.9%, 48.2%, 37.0%, 28.8% and 47.9% of all nurses. Frontline nurses reported experiencing more moderate to severe symptoms of depression, distress and burnout. Furthermore, females reported more burnout as compared to males. Multivariate logistic regression analysis showed that after adjustment, working in the frontlines was an independent risk variable for depression and burnout.

**Conclusion:**

This is one of the few studies looking at mental health outcomes among nurses during the COVID-19 pandemic in Kenya. Similar to other studies from around the world, nurses directly involved with COVID-19 patients reported higher rates of mental health symptoms. Burnout threatens to exacerbate the pre-existing severe nursing workforce shortage in low-resource settings. Cost-effective and feasible mitigating strategies, geared to low-middle income countries, are urgently needed to help cope with mental health symptoms during such a pandemic.

## Introduction

According to the World Health Organization (WHO), Kenya, in 2020, confirmed over 95,000 COVID-19 cases with about 1,655 deaths [1]. Healthcare workers historically play a critical role in addressing the medical and psychological needs of patients during a pandemic. A robust healthcare system is required to adequately attend to the mental well-being needs of not only the patients, but healthcare workers too.

The Kenyan health system is defined by the following 6 levels of care provided to patients: level 1: community services, level 2: dispensaries and clinics, level 3: health centres, level 4: sub-county hospitals, level 5: county referral hospitals and large private hospitals and level 6: national referral hospital and large private teaching hospitals. Kenya’s health budget accounts for 4% of the total budget and the funds are primarily used for curative services. Government funds are solely distributed to the sub-county and government clinical facilities and hospitals [2]. In Kenya, the total number of healthcare workers employed by public as well and private institutions is approximately 31,412 as per the 2016 Training Needs Assessment. This number remains way below the required 138,266 healthcare workers suggested by the Ministry of Health’s Norm and Standard Guidelines [3].

Nurses remain a critical part of the work force in Kenya often on the frontline providing initial and key services to patients. There are approximately 51,649 nurses below the age of 60 with majority being female between the ages of 21–30 years. Majority of the nursing training institutions are government sponsored and may offer three levels of training programs including: certificate, diploma and a bachelor’s degree. A majority of the nurses (65%) in Kenya are diploma holders [4]. Multiple studies have consistently reported an overall shortages of nurses in Kenya especially in the public sector countrywide with major variations in nurse densities and skills around the various geographic areas in Kenya [3, 5].

Prior to the COVID-19 pandemic, the Ebola pandemic nearly demolished an already fragile healthcare system in West Africa. Healthcare workers were 20–30 times more likely than the general population to contract the virus, and mortality among healthcare workers was reported as high as 69% [6, 7]. Despite these alarming facts, little was published on the effects of the Ebola pandemic on the mental health and well-being of healthcare workers in West Africa. Similarly, the COVID-19 pandemic in sub-Saharan Africa (SSA) continues to pose many challenges to the mental well-being of healthcare workers. This is augmented by the fact that many countries in SSA have limited resources including healthcare workers, intensive care units (ICUs) and ventilators to deal with the increasing number of COVID-19 patients. SSA has approximately 11% of the world population and 24% of the global disease burden but on 3% of the world’s healthcare workers [8]. WHO in 2018 reported approximately 0.2 physicians and 1.2 nurses per 1,000 population in Kenya, in contrast to 2.6 physicians and 11.9 nurses per 1,000 people in the United States [9, 10]. The limited health care resources and increasing workload within a fragile health care system exacerbate the mental health strain faced by many healthcare workers, especially nurses, taking care of COVID-19 patients.

The pandemic has been linked to various psychological disorders especially in healthcare workers involved in the direct care of COVID-19 patients, with many studies showing increased levels of depression, anxiety, stress and burnout most notably among frontline nurses [11–18]. A study looking at the prevalence of mental health disorders among nurses in China during the COVID-19 outbreak, found that COVID-19 related stress (such as workload and fear of infection) was associated with higher risk of depression and anxiety. A similar study looking at mental health impact among health workers in a low resource setting (Nepal) during the COVID-19 pandemic also found significant levels of depression, anxiety and insomnia. Nurses showed a higher level of anxiety when compared to other healthcare workers [19]. Furthermore, a survey of 502 healthcare workers in Saudi Arabia during the COVID-19 outbreak similarly showed higher levels of anxiety among the nurses while a study looking at Canadian critical care nurses involved in the early phase of the pandemic also found significant symptoms of post-traumatic stress, depression and anxiety related to taking care of COVID-19 patients [20, 21].

Unfortunately, one year into the pandemic, little is known about the effects of COVID-19 on mental well-being of healthcare workers especially nurses in SSA. Due to the paucity in data and limited resources, including mental health care in sub-Saharan Africa when compared to western countries, we sought to explore the mental wellbeing of nurses taking care of COVID-19 patient at a tertiary healthcare facility in Kenya.

## Methods

We carried out a cross sectional survey between August and November, 2020. Email invitations with a link to a voluntary, de-identified survey was sent to all the nurses at the Aga Khan University Hospital, Nairobi (AKUHN). Email reminders were sent out twice a week from REDCap for participation in the survey. The link to the survey could only be used once. Responses from the nurses remained anonymous, Approval for this study was obtained from the Institutional Ethics and Review Committee AKHUN and online survey data was collected through the Research Electronic Data Capture—REDCap platform (Vanderbilt and National Institute of Health) [22].

There was a steady increase in COVID-19 cases from October into November. Kenya recorded the highest number of cases in a single month (~29,000 confirmed cases, ~470 deaths) in November 2020. Based on the 7-day average from John Hopkins University data dashboard, the peak of the first wave occurred in early August whereas the peak of the second wave occurred in mid-November in Kenya [23].

### Data collection

The survey questionnaire, in English, consisted of demographic characteristics, job title, experiences providing care to patients with COVID-19, symptoms of depression, anxiety, insomnia, distress and burnout. The mental health symptoms were measured using validated questionnaires that would allow for comparison across different populations: the 9-item Patient Health Questionnaire (PHQ-9) [24], the 7-item Generalized Anxiety Disorder Questionnaire (GAD-7) [25], the 7-item Insomnia Severity Index Questionnaire (ISI) [26], the 22-item Impact of Event Scale–Revised (IES-R) [27] and the 16-item Stanford Professional Fulfillment Index Questionnaire (SPFI) [28]. The cut-off score for detecting symptoms of major depression, anxiety, insomnia, and distress were 10, 7, 15, and 26 respectively [16]. On conclusion of the survey, a score for each mental health disorder was computed and shared with the participant. If the scores indicated a mental health issue such as mild moderate or severe symptoms or any suicidal thoughts, the participants were directed to seek medical consultation with their primary care provider or the counselling services at the hospital. A hotline was specifically available for all participants with urgent mental health related to the COVID-10 pandemic. In addition, participants were encouraged to contact the principal investigator for any questions or concerns regarding their survey scores or unavailability to access mental health services.

### Statistical analysis

Categorical data was presented as frequencies and percentages whereas continuous data was presented as medians with interquartile ranges (IQR). Normality tests were employed using the Shapiro-Wilk test. Kruskal-Wallis test and Fisher’s Exact test was utilized to compare the continuous and categorical variables. Logistic regression analysis was performed, and the associations between risk factors and outcomes are presented as odds ratios (ORs) and 95% CIs, after adjustment for confounders, including age, gender, and marital status. Data analysis was performed using SPSS statistical software version 20.0 (IBM Corp). The significance level was set at α = .05, and all tests were 2-tailed.

## Results

Between August 2020 and November 2020, 255 nurses from AKUHN were invited to participate in the study and 171 (67.1%) consented to complete the survey. The median age of the participants was 33.47 years (IQR: 29.80–35.82). Majority of the nurses were females (70.2%) and married (60.8%). A total of 111 nurses (64.9%) were frontline workers directly engaged in COVID-19 care. Majority of the nurses (64.0%) had cared for about 5–20 patients and 66.7% had lost a patient to COVID-19. Approximately, 60.0% of the nurses reported having enough resources to care for COVID-19 whereas 71.2% reported to have been adequately trained for COVID-19. Only 1.8% had reported a prior history or diagnosis of any mental health disorder.

Depression, anxiety, insomnia, distress, and burnout were reported (mild to severe) in 45.9%, 48.2%, 37.0%, 28.8% and 47.9% of all nurses. Frontline nurses reported experiencing more moderate to severe symptoms of all the mental health disorders than the second line nurses. Moderate to severe; depression among frontline vs second line nurses was 17.3% vs 3.4% (p = 0.026); anxiety among frontline vs second line nurses was 11.1% vs 5.1% (p = 0.681); insomnia among frontline vs second line nurses was 9.1% vs 0.0% (p = 0.052); distress among frontline vs second line nurses was 20.4% vs 11.9% (p = 0.039); burnout among frontline vs second line nurses was 56.1% vs 32.8% (p = 0.005) (Table 1). Continuous data such as age and the mental health scores were not normally distributed where the p values based on Shapiro-Wilk test was all < 0.05. The questionnaires demonstrated good internal consistency with a Cronbach alpha of 0.835, 0.837, 0.871, 0.955 and 0.837 for PHQ-9, GAD-7, ISI, IES-R and SPFI respectively.

**Table 1:** Comparison of demographic and mental health disorders among the frontline and second line nurses.

	*Total*	*Frontline*	*Secondline*	*P value*
Age (n = 164) (median [IQR])	33.47 [29.80, 35.32]	33.49 [29.52, 36.09]	33.30 [29.97, 35.81]	*0*.*817*
***Gender***				*0*.*741*
Male	50 (29.2%)	34 (30.6%)	16 (26.7%)	
Female	120 (70.2%)	76 (68.5%)	44 (73.3%)	
Prefer not to disclose	1 (0.6%)	1 (0.9%)	0 (0.0%)	
***Marital Status***				*0*.*506*
Single	61 (35.7%)	37 (33.3%)	24 (40.0%)	
Married	104 (60.8%)	69 (62.2%)	35 (58.3%)	
Other	6 (3.5%)	5 (4.5%)	1 (1.7%)	
***History of Mental Health Disorder***				*0*.*553*
Yes	3 (1.8%)	3 (2.7%)	0 (0.0%)	
No	168 (98.2%)	108 (97.3%)	60 (100.0%)	
***Depression***		**(n = 110)**	**(n = 60)**	*0*.*026*
None	92 (54.1%)	52 (47.3%)	50 (66.7%)	
Mild	57 (33.5%)	39 (35.5%)	18 (30.0%)	
Moderate	12 (7.1%)	11 (10.0%)	1 (1.7%)	
Severe	9 (5.3%)	8 (7.3%)	1 (1.7%)	
Score	4.00 [2.00, 7.00]	5.00 [2.00, 9.00]	3.00 [1.00, 6.00]	*0*.*009*
***Anxiety***		**(n = 109)**	**(n = 59)**	*0*.*681*
Minimal	87 (51.8%)	55 (50.5%)	32 (54.2%)	
Mild	66 (39.3%)	42 (38.5%)	24 (40.7%)	
Moderate	12 (7.1%)	9 (8.3%)	3 (5.1%)	
Severe	3 (1.8%)	3 (2.8%)	0 (0.0%)	
Score	4.00 [2.00, 7.00]	4.00 [2.00, 7.00]	4.00 [2.00, 6.00]	*0*.*383*
***Insomnia***		**(n = 109)**	**(n = 59)**	*0*.*052*
None	106 (63.1%)	69 (63.3%)	37 (62.7%)	
Subthreshold	52 (31.0%)	30 (27.5%)	22 (37.3%)	
Moderate	9 (5.4%)	9 (8.3%)	0 (0.0%)	
Severe	1 (0.6%)	1 (0.9%)	0 (0.0%)	
Score	6.00 [2.00, 9.00]	6.00 [1.00, 9.00]	6.00 [3.00, 9.00]	*0*.*533*
***Distress***		**(n = 108)**	**(n = 59)**	*0*.*039*
Normal	119 (71.3%)	74 (68.5%)	45 (76.3%)	
Mild	19 (11.4%)	12 (11.1%)	7 (11.9%)	
Moderate	9 (5.4%)	4 (3.7%)	5 (8.5%)	
Severe	20 (12.0%)	18 (16.7%)	2 (3.4%)	
Score	15.00 [6.00, 26.00]	16.00 [5.00, 28.50]	14.00 [7.00, 23.00]	*0*.*501*
Avoidance	0.90 [0.30, 1.40]	0.90 [0.30, 1.70]	0.70 [0.30, 1.10]	*0*.*205*
Intrusion	0.80 [0.30, 1.30]	0.80 [0.20, 1.30]	0.80 [0.40, 1.30]	*0*.*900*
Hyperarousal	0.50 [0.20, 1.00]	0.50 [0.20, 1.20]	0.50 [0.20, 0.80]	*0*.*619*
***Burnout***		**(n = 107)**	**(n = 58)**	*0*.*005*
≤ 1.33	86 (52.1%)	47 (43.9%)	39 (67.2%)	
> 1.33	79 (47.9%)	60 (56.1%)	19 (32.8%)	
***Professional Fulfillment***		**(n = 107)**	**(n = 57)**	*0*.*298*
≤ 3.00	133 (81.1%)	84 (78.5%)	49 (86.0%)	
> 3.00	31 (18.9%)	23 (21.5%)	8 (14.0%)	
*Professional Fulfillment*	54.17 [41.67, 75.00]	54.17 [37.50, 75.00]	54.17 [45.83, 70.83]	*0*.*967*
*Work Exhaustion*	50.00 [25.00, 62.50]	50.00 [25.00, 75.00]	37.50 [25.00, 56.25]	*0*.*011*
*Interpersonal Disengagement*	16.67 [0.00, 37.50]	25.00 [0.00, 37.50]	12.50 [0.00, 25.00]	*0*.*027*

* N was not the same down the column because variable availability of results.

On comparing the differences between gender and mental health symptoms, females reported experiencing more symptoms of all the mental health disorders than males. However, only burnout was found to be statistically significantly higher in females (female vs male nurses: 53.9% vs 34.0% (p = 0.027)) (Table 2).

**Table 2:** Comparison of mental health disorders among the male and female nurses.

	*Males*	*Females*	*P value*
***Depression***	**(n = 50)**	**(n = 120)**	*0*.*323*
None	26 (52.0%)	66 (55.0%)	
Mild	21 (42.0%)	36 (30.0%)	
Moderate	2 (4.0%)	10 (8.3%)	
Severe	1 (2.0%)	8 (6.7%)	
***Anxiety***	**(n = 50)**	**(n = 118)**	*0*.*516*
Minimal	30 (60.0%)	57 (48.3%)	
Mild	17 (34.0%)	49 (41.5%)	
Moderate	3 (6.0%)	9 (7.6%)	
Severe	0 (0.0%)	3 (2.5%)	
***Insomnia***	**(n = 50)**	**(n = 118)**	*0*.*214*
None	37 (74.0%)	69 (58.5%)	
Subthreshold	12 (24.0%)	40 (33.9%)	
Moderate	1 (2.0%)	8 (6.8%)	
Severe	0 (0.0%)	1 (0.8%)	
***Distress***	**(n = 50)**	**(n = 117)**	*0*.*063*
Normal	39 (78.0%)	80 (68.4%)	
Mild	8 (16.0%)	11 (9.4%)	
Moderate	1 (2.0%)	8 (6.8%)	
Severe	2 (4.0%)	18 (15.4%)	
***Burnout***	**(n = 50)**	**(n = 115)**	*0*.*027*
≤ 1.33	33 (66.0%)	53 (46.1%)	
> 1.33	17 (34.0%)	62 (53.9%)	
***Professional Fulfillment***	**(n = 49)**	**(n = 115)**	*0*.*514*
≤ 3.00	38 (77.6%)	95 (82.6%)	
> 3.00	11 (22.4%)	20 (17.4%)	

* N was not the same down the column because variable availability of results.

Multivariate logistic regression analysis showed that after adjustment, working in the frontlines was an independent risk variable for depression and burnout (depression, OR 5.98; 95% CI, 1.31–27.33; p = 0.021; burnout: OR, 3.37, 95% CI, 1.63–6.97) (Table 3).

**Table 3:** Multivariate logistic regression analysis.

*Variable*	*Adj OR*	*95% CI*	*P value*
***PHQ-9*: *Depression***			
Covid Care: Frontline	5.98	1.31–27.33	0.021
***GAD-7*: *Anxiety***			
Covid Care: Frontline	2.20	0.58–8.33	0.247
***IES-R*: *Distress***			
Covid Care: Frontline	1.95	0.88–4.32	0.098
***SPFI*: *Burnout***			
Covid Care: Frontline	3.37	1.63–6.97	0.001
***SPFI*: *Professional Fulfillment***			
Covid Care: Frontline	1.72	0.70–4.22	0.234

* Adjusted for age, gender, and marital status.

Adj OR: adjusted odds ratio, CI: confidence interval.

## Discussion

To the best of our knowledge, this is the first study of its kind looking at the mental wellbeing of nurses taking care of COVID-19 patient in a tertiary healthcare facility in Kenya. Comparable to other studies, we found over 40% of front-line nurses at our facility who took care of COVID-19 patients suffered from mild to severe depression [14, 29, 30]. Only 1.8% of the participants in our study reported ever having mental health disorders previously. To help put this into perspective, prior to COVID-19, the WHO on world mental health situation placed Kenya as the sixth most depressed country in Africa with a depression rate of 4.4% [31], while a recent study conducted by Nyongesa and colleagues found a prevalence of 13.8% of depressive symptoms in adults living with HIV in rural Kilifi in Kenya [32].

Our study also aligns with a number of other studies showing that front-line nurses, especially females, suffer from poor sleep, depression and anxiety [11, 15, 33, 34]. Furthermore, similar to a study conducted by Huang and colleagues, we also found that females nurse at our institution showed higher rates of all mental disorders compared to their male counterparts [15].

Tu and colleagues looked at sleep quality and mood symptoms in front-line nurses in Wuhan, China during the COVID-19 outbreak and found that 60% had poor sleep, 46% suffered depression symptoms and 40% reported anxiety symptoms [11]. Similarly, Zhan and colleagues reported a prevalence of insomnia in 52.8% of the frontline nurses in Wuhan, China during the COVID-19 pandemic [33]. In our study, front-line nurses had higher scores for moderate to severe anxiety and insomnia, but these were not statistically significant when compared to second-line nurses who were not involved in care of COVID-19 patients. Our results could potentially have been affected by the smaller size of our study population as compared to other studies.

Similar to other studies [12, 35–37], we also found that front-line nurse at our institution were more prone to burnout compared to second-line nurses who were not involved in the care of COVID-19 patients. Hu and colleague reported that 50% of nurses in their study reported moderate to high burnout with regards to emotional exhaustion, depersonalization and personal accomplishment [35]. We also found that female nurses at our institution expressed more burnout than their male counterparts. This is not unique to our study as other studies have also shown that female healthcare workers, especially nurses are more prone to psychological disorders, including burnout due to the COVID-19 pandemic [16]. More importantly, burnout in nurses has been linked to an increase in turnover rates which can have major implications in countries with an already fragile healthcare work force and system [38].

Shechter and colleagues in their study looking a psychological distress among New York healthcare workers during the COVID-19 pandemic found 57% of their participants screened positive for acute stress. In addition, nurses and advanced practice providers had a higher percentage of psychological symptoms compared to attending physicians [39]. In contrast, Liu and colleagues in their study looking at psychological impact of COVID-19 on nurses in China found second line nurses had a higher incidence of distress than frontline nurses [13]. In our study, we found that front-line nurses at our institution had higher rates of distress compared to second-line nurses not involved in the care of COVID-19 patients. However, we did not find any significant association between sociodemographic variable age or marital status and mental well-being of nurses at our facility. This is contrary to what other studies have found and our findings could have been influenced by our study population size.

Our study has several limitations. All the participants were from one institution and this limits the generalization of our findings to other healthcare institutions in Kenya. In addition, psychological outcomes among nurses could certainly have been more pronounced if the study had been conducted in a larger cohort for a longer duration. Thirdly, an online survey is often skewed by a selective population that is technologically familiar with the use of internet and email. Finally, even though our response rate was 67%, response bias may still exist especially if the nurses who did not respond were either too stressed to respond or not stressed at all to respond to the survey.

## Conclusion

The current pandemic is weighing heavily on our healthcare workforce. In Kenya, this burden is particularly concerning given the acute shortage of nurses’ even prior to the pandemic. As many countries in SSA struggle with limited medical resources and fragile health care systems amidst the COVID-19 pandemic, the mental health burden on all healthcare workers especially in resource-limited settings must be appropriately measured and addressed. Our study sheds light on the mental well-being among nurses in Kenya and similar to other studies reflects on the psychological burden that frontline nurses are encountering during this pandemic. However, further research to understand the long-term effects of COVID-19 on the mental well-being of nurses, especially in sub-Saharan Africa, are warranted. Our study has major implications including the need for cost-effective and easy to implement strategies across the healthcare system to support the mental well-being of healthcare workers especially nurses.

## Supporting information

S1 File (XLSX)
